# Ptychography: A brief introduction

**DOI:** 10.1111/jmi.70025

**Published:** 2025-08-20

**Authors:** John Rodenburg

**Affiliations:** ^1^ School of Electrical and Electronic Engineering The University of Sheffield Sheffield UK

**Keywords:** computational imaging, diffraction, high resolution, lensless imaging, multiple scattering, ptychography

1

For anyone new to ptychography, the first obstacle to overcome is how to pronounce its name. The author has heard many tortured attempts trying to simultaneously incorporate the ‘p’ with the ‘t’—an impossible task. The answer is very simple: forget the ‘p’—in English it is silent, just as in ‘psychology’. Pronounce it as ‘tykography’.

Ptychography overcomes the two most enduring historical weaknesses of conventional transmission (and reflection) microscopy. It can in principle obtain wavelength limited resolution, unaffected by lens aberration or the maximum scattering angle imposed by the numerical aperture of the lens. This is especially important for X‐ray and electron imaging where, for various intractable reasons, the useable numerical aperture of the available lenses is so small. It can also record the image phase near perfectly, meaning that otherwise transparent objects can be imaged with very high contrast.

Unlike conventional microscopy with lenses, ptychography does not provide a real or virtual image that can be seen directly. Instead, it uses a computer to process a very large quantity of data that bear no relationship to the final image that it ‘reconstructs’. Ordinary microscopists—that is, those who simply want to see a magnified image of their specimen and do not want to understand exactly how the image is computed—can find this circuitous process all rather alienating. First results from the author's group in the early 1990s were widely dismissed by the community. A leading microscopist at the time asserted that he would never believe in an image that came out of a computer. A further problem was that the pictures we could obtain in those days were so small and totally unconvincing. Ptychography had to wait for Moore's Law to catch up with its greedy data requirements.

However, in the last 10–15 years, ptychography has become the technique of choice for very high‐resolution X‐ray imaging and tomography. In the last 5 years or so, some extraordinary electron ptychography results have been reported, far surpassing the resolution limit that for so many years had seemed insurmountable using magnetic lenses and aberration correction. Optical microscopy is already wavelength limited, but the very sensitive phase image that ptychography supplies has removed the need for staining or labelling, thus allowing live imaging of biological cells.

The experimental method is deceptively simple. We have a source of radiation which shines upon the specimen. The wavefield at the exit surface of this specimen is then allowed to propagate some distance downstream of the object where the pattern of scattered intensity is recorded on a two‐dimensional detector. It is important to understand that this detector can be as large as we like. It can capture scattering up to large angles, where high‐resolution information is expressed. Electron and X‐ray lenses can only capture and focus reliably small angles of scatter, which severely limits their resolution.

We then arrange for the specimen and the illumination to be moved laterally relative to one another, whereupon the scattered intensity is recorded again. The process is repeated several times (in practice, this can be as many as 100 or 1000 times) in such a way that each area of interest of the specimen is illuminated at least once. A necessary condition for the computation of the image is that the area of specimen illuminated at any one position must also overlap with at least one other area of the specimen which has also been illuminated.

This overlap is important because it means that the same element (pixel) of the specimen is expressed in more than one scattering pattern, meaning that we have redundancy in the data: we record many more data than the number of numbers we need to compute the final image. These ‘extra’ data are of fundamental importance in ptychography.

First, to make an image, we must solve the ‘phase problem’. Every measurement we make—every pixel in every scattering pattern (usually a diffraction pattern)—can only be recorded in intensity. However, the underlining wave impinging on the detector has two numbers associated with it: a modulus and a phase or, equivalently, the real and imaginary components of a complex number. In some imaging techniques, like radio astronomy, the frequency of the wave disturbance is low enough so that we can measure its amplitude and its time of arrival (which is encoded in the phase) directly, say by plotting the signal on a cathode ray tube. This is as much as we can ever measure about a propagating wave. If we assemble all the data from many detectors, then we can work out backwards the shape of the source of the waves: that is, an image of the object.

However, to see very small objects, we need to use a radiation with a wavelength concomitant with the size of that object, which itself implies a very high frequency wave. For the microscopic radiations (light, X‐ray and electrons), there are no detectors that can record directly the phase of such waves: only the intensity (the modulus squared) can be measured. All phase information is lost.

The genius of ptychography is that it recovers this ‘lost phase’ by exploiting the effect that the lateral shift of the illumination/specimen has on the recorded data. Once we have solved for the phase of the wave over the entire detector, we can use this to generate a computational lens which has a much larger numerical aperture (and hence can achieve much higher resolution) than the very small numerical apertures achieved by short wavelength (X‐ray and electron) lenses.

An essential mathematical constraint is that the two functions that move across one another remain constant during the course of the experiment. However, there is great flexibility in the physical nature of the functions themselves. For example, in Fourier ptychography, one of the functions is the wavefield lying in the back focal plane of a low‐resolution microscope, while the other function is the objective aperture lying in the same plane. Tilting the illumination has the effect of shifting the wavefield pattern across the aperture. In this case, the data collection occurs in the image plane which lies in the Fourier domain of the aperture.

Although nowadays taken for granted, it is not at all obvious that the illumination/specimen shifts used in ptychography should allow for the solution of the phase problem. We can argue that the data set we record is highly constrained because of the overlaps between illumination positions. But then does it automatically follow that the relative phases of all the diffraction patterns can be solved for unambiguously? When the author first considered this issue in the late 1980s, the answer was far from clear. It was at that time that Owen Saxton (as of the Gerchberg and Saxton phase retrieval algorithm) suggested that he might consider looking at some work by Walter Hoppe from the late 1960s and early 1970s. This had shown that moving a carefully designed coherent illumination field across a crystal specimen could, in theory, solve for the phase difference between adjacent crystalline reflections. The method was demonstrated using light and a one‐dimensional grating. Hegerl and Hoppe later referred to the scheme as ‘ptychography’ because it required the diffracted beams to be convolved or ‘folded’ into one another. ‘Ptych’ is the ancient Greek for ‘fold’. (Incidentally, it also means—amongst other things—the entrails of an animal and the folds in gently rolling hills).

For the author, this was a pivotal insight. If relative phases could be found for pairs of diffracted beams, then surely this same concept—moving an illumination field—could be extended to general, non‐crystalline objects, hence solving for the phases between all such pairs of beams? For an extended non‐crystalline specimen, the diffraction pattern involves interferences between millions of diffracted beams. Nevertheless, it is useful to have a simple model for why ptychography should in principle be able solve the phase problem. Unfortunately, because the original papers written by Hoppe are difficult to understand (and are in German), those new to the field often find the ‘ptych’ concept rather confusing and irrelevant.

Today, nobody does ptychography in the way it was initially envisaged. Its original applicability is extremely narrow: perfect crystal structures can be easily solved using X‐ray methods so there is no real scientific need for ptychography of crystals. However, in the mid‐1990s, electron crystalline ptychography in its original form—interfering pairs of beams in the scanning transmission electron microscope (STEM) configuration—was indeed shown to work.

A much more difficult problem is to reconstruct the specimen function for some general ptychographical data set—that is, one where the data have been scattered from an infinite (and possibly 3D) object which has complicated non‐crystalline structure, and the form of the illumination is unknown.

Nowadays nearly all reconstruction algorithms converge upon a solution iteratively. We assume we know the way that the illumination interacts with the specimen and how the resulting scattered wave propagates to the detector. This might have to include modelling scattering from multiple layers of a thick specimen. Indeed, it was a major advance in ptychography to realise that its data could be used to solve for 3D structures. At any particular iteration, we have an ongoing estimate of the specimen function and the illumination function. We then calculate the intensity of the set of diffraction patterns we would expect these functions to generate. Of course, if our estimated functions are not the same as the as their actual counterparts, the modelled data will not be the same as the real data. We use this difference to guide us to a new estimate of the specimen and illumination functions and then repeat the process iteratively until the real and the estimated data match with one another. There are a great number of ways to implement this sort of scheme in practice.

We also mention that there are two non‐iterative ‘direct’ inversion methods which were developed during the 1990s: the Wigner Distribution Deconvolution (WDD) and the single sideband method (SSB). These are still being used by some workers. They have some distinct advantages (and also some limitations), but we do not have space to describe them here.

The first iterative approach to the reconstruction problem (called the ptychographic iterative engine, ‘PIE’) was published 2004. This was shown to work experimentally with hard X‐rays in 2007 and subsequently led to an explosive interest in ptychography at X‐ray synchrotrons around the world. Although ptychography applies to any wavelength, there were a number of reasons why it made such a large and immediate impact in the field of X‐ray imaging. First, the gain in resolution beyond the capabilities of a typical X‐ray lens was by about a factor of 5. Second, the phase image provided by ptychography is ideal for tomography; phase is cumulative as it passes through the object, and so it gives a linear measure of how much material density the beam has passed through. X‐ray ‘ptycho‐tomography’ is now a standard technique at many beamlines. Thirdly, the single photon‐counting hard X‐ray detectors available were much more efficient than any electron detector at that time. Ptychography had come of age.

Part of the beauty of ptychography is that the mathematics of the reconstruction process can be applied to any wavelength of microscopic imaging. Once the inverse problem had been solved, it quickly found applications in light, electron, EUV, and Terahertz imaging.

As mentioned above, ptychography relies on redundancy in the data we record to solve the phase problem. In fact, in certain experimental configurations, this redundancy can be huge: we can in principle obtain a 2D diffraction pattern for every single pixel in the (2D) object/image plane. In electron imaging, this is now referred to as ‘4D STEM’. If we record a 4D data set to solve for a 2D image, we clearly have a super‐abundance of this ‘extra’ data. We can use this redundancy to greatly enhance the capabilities of the technique. There is no space here to describe all the advances in the technique that use these ‘extra’ data. Suffice it to say that key developments over the last 10–15 years have included methods for removing partial coherence in the source and illumination optics, methods for coping—and indeed computationally reversing—3D multiple scattering effects (important in electron imaging, where scattering is very strong), and methods to retrospectively correct errors that occurred during the data collection.

But the story is far from over. There is still a lot of work to do to make ptychography as easy to use as a conventional microscope. It is now seen as a standard technique at dedicated X‐ray beamlines, but even then, a user needs a lot of understanding of the technique to optimise results. Electron ptychography is much more difficult and is a very long way off from being accessible to non‐specialists, even though it holds great promise: the increased resolution it provides has made it possible to image for the first time atomic vibrations and the bonding of atoms.

So, ‘ptychography’ is an irritating word for a technique that is revolutionising microscopy over all wavelengths, both photon and electron. Its capabilities continue to expand very quickly. Ironically, modern incarnations of it bear little or no relationship to the original concept described by its name: but the word is now baked into the literature. The applications of ptychography are so wide it would be very hard to come up with a single term that could include all its incarnations. I think we must learn to live with this wretched name forever: but whatever you do, please don't try to pronounce that ‘p’!

